# Towards a consensus definition of maternal sepsis: results of a systematic review and expert consultation

**DOI:** 10.1186/s12978-017-0321-6

**Published:** 2017-05-30

**Authors:** Mercedes Bonet, Vicky Nogueira Pileggi, Marcus J Rijken, Arri Coomarasamy, David Lissauer, João Paulo Souza, Ahmet Metin Gülmezoglu

**Affiliations:** 1 UNDP/UNFPA/UNICEF/WHO/World Bank Special Programme of Research, Development and Research Training in Human Reproduction (HRP), Department of Reproductive Health and Research, World Health Organization, Geneva, Switzerland; 2 Department of Social Medicine and Department of Paediatrics, Ribeirão Preto Medical School, University of São Paulo, Ribeirão Preto, SP Brazil; 3 Department of Obstetrics and Gynaecology and Julius Global Health, Julius Center for Health Sciences and Primary Care, Utrecht University Medical Centre, Utrecht, The Netherlands; 4 Institute of Metabolism and Systems Research, University of Birmingham, Birmingham, UK; 5 Birmingham Women’s National Health Service (NHS) Foundation Trust, Birmingham, UK; 6 Birmingham Centre for Women’s and Children’s Health, College of Medical and Dental Sciences, University of Birmingham, Birmingham, UK

**Keywords:** Sepsis, Maternal sepsis, Consensus, Definition

## Abstract

**Background:**

There is a need for a clear and actionable definition of maternal sepsis, in order to better assess the burden of this condition, trigger timely and effective treatment and allow comparisons across facilities and countries. The objective of this study was to review maternal sepsis definitions and identification criteria and to report on the results of an expert consultation to develop a new international definition of maternal sepsis.

**Methods:**

All original and review articles and WHO documents, as well as clinical guidelines providing definitions and/or identification criteria of maternal sepsis were included. A multidisciplinary international panel of experts was surveyed through an online consultation in March-April 2016 on their opinion on the existing sepsis definitions, including new definition of sepsis proposed for the adult population (2016 Third International Consensus Definitions for Sepsis and Septic Shock) and importance of different criteria for identification of maternal sepsis. The definition was agreed using an iterative process in an expert face-to-face consensus development meeting convened by WHO and Jhpiego.

**Results:**

Standardizing the definition of maternal sepsis and aligning it with the current understanding of sepsis in the adult population was considered a mandatory step to improve the assessment of the burden of maternal sepsis by the expert panel. The literature review and expert consultation resulted in a new WHO consensus definition “Maternal sepsis is a life-threatening condition defined as organ dysfunction resulting from infection during pregnancy, child-birth, post-abortion, or post-partum period”. Plans are in progress to validate the new WHO definition of maternal sepsis in a large international population.

**Conclusion:**

The operationalization of the new maternal sepsis definition requires generation of a set of practical criteria to identify women with sepsis. These criteria should enable clinicians to focus on the timely initiation of actionable elements of care (administration of antimicrobials and fluids, support of vital organ functions, and referral) and improve maternal outcomes.

**Electronic supplementary material:**

The online version of this article (doi:10.1186/s12978-017-0321-6) contains supplementary material, which is available to authorized users.

## Plain english summary

Sepsis occurs when the body’s response to infection damages its own organs and tissues. If not recognised early and treated timely, sepsis can progress to shock and death. Physiological, immunological and mechanical changes in pregnancy make pregnant women more susceptible to infections compared with non-pregnant women. Furthermore, physiological adaptations to pregnancy may obscure signs and symptoms of infection and sepsis. Efforts have been made in the last 25 years to standardize definitions of sepsis for the general adult population, but the validity and applicability of the proposed definitions and identification criteria to pregnant women are uncertain.

We reviewed articles and WHO documents, as well as clinical guidelines providing definitions and/or identification criteria of maternal sepsis. We also surveyed a multidisciplinary international panel of experts on their opinion on the existing sepsis definitions and importance of different criteria for identification of maternal sepsis. The literature review and expert consultation resulted in a new WHO consensus definition. Plans are in progress to validate the new WHO definition of maternal sepsis and identification criteria in a large international population. These identification criteria should enable clinicians to focus on the timely initiation of actionable elements of care (administration of antimicrobials and fluids, support of vital organ functions, and referral) and improve maternal outcomes.

## Introduction

Sepsis is a major public health concern [1]. Sepsis includes three essential components: infection, host response to infection and organ dysfunction. A wide range of pathogens could cause life threatening responses in many organs; consequently sepsis has a broad spectrum of clinical presentations. Pregnant women are particularly predisposed to develop infections and sepsis for several reasons. Physiological, immunological and mechanical changes in pregnancy make pregnant women more susceptible to infections compared with non-pregnant women, particularly during the postpartum period [2]. Furthermore, physiological adaptations to pregnancy (e.g. hyperdynamic circulation, tachycardia, diminished oxygen reserve, hypercoagulability), maternal efforts during second stage of labour, interventions during labour, or blood loss, may obscure signs and symptoms of infection and sepsis [3, 4]. This may result in delays in the recognition and treatment of sepsis [5]. Another factor to be considered is that a substantial proportion of early onset neonatal sepsis originates from intra-uterine infections and antepartum maternal infections [6, 7].

Globally, important achievements in healthcare (for example, hand washing, antibiotics) [8] have had a great impact on the reduction of infection-related maternal deaths; however sepsis is still an important contributor to preventable maternal mortality. Infections are considered the underlying cause in about 11% of maternal deaths [9] and are a significant contributor to many deaths attributed to other conditions. However, the true frequency of maternal infections and its complications are not well known. Imprecise and varying definitions may have led to discrepancies in reported incidence and observed mortality [1, 2, 10]. Available data on pregnancy associated sepsis from high-income countries report an incidence of 9 to 49 per 100,000 deliveries-year, depending on the definition and population used [11]. Lack of data from low-income countries makes the incidence difficult to determine in those countries [12]. Compared to other pregnancy complications the case fatality rate of maternal sepsis is very high. Sepsis with acute organ dysfunction has a mortality of 20–40% in high-income countries in the early 2000s [13], but more recent data shows an overall rate of 8% [14], and 14% in women with septic shock [15]. Estimates of case fatality rates after puerperal infection vary from 4 to 50% in Africa and Asia [16].

Efforts have been made in the last 25 years to standardize definitions of sepsis for the general adult population [1, 17–19]. In 2016, definitions and clinical criteria for identifying sepsis and septic shock were updated [1] and the Sequential [Sepsis-related] Organ Failure Assessment (SOFA) score was proposed to classify organ dysfunction and thus identify sepsis in the adult population [1]. However, the validity and applicability of the proposed definition and identification criteria to pregnant women are uncertain.

The objective of this paper is to report the development of a standard definition for maternal sepsis based on a systematic review of literature and a technical consultation.

## Materials and methods

The development of a consensus definition of maternal sepsis was based on two components, a systematic review of literature and a technical consultation.

### Systematic review

The systematic review of literature focused on definitions and identification criteria of maternal sepsis. Prognostic literature, including articles on prediction scores used to identify obstetric patients at risk of developing complications, were not included in this review.

#### Search strategy

We conducted an electronic search to identify review articles on maternal sepsis and original articles reporting on the development and testing of maternal sepsis definitions or identification criteria for maternal sepsis. Clinical guidelines and WHO documents related to the prevention, identification and management of maternal sepsis were also reviewed. The literature search in PubMed/MEDLINE and EMBASE databases was carried out on 15/02/2016 and included papers published from 01/01/2010 to 15/02/2016. The search strategy used a combination of the following terms, expanded and adapted for each database: “sepsis”, “septicemia”, “septic shock”, “maternal”, “mother”, “pregnancy”, “childbirth”, “postpartum”, “death”, “mortality”, “severe morbidity”, “critical illness”, “near-miss”, “intensive care”, “critical care”, “emergency”, “definition”, “identification criteria”, and “diagnostic criteria”. Additional details of the search strategy are provided in the Additional file 1: Table S1. Guidelines and WHO documents related to the prevention, identification and treatment of maternal sepsis were searched using the terms “maternal sepsis” and “guideline” in the following databases: National Guideline Clearinghouse, WHO guidelines repository, Guidelines International Network (G-I-N), National Institute for Health and Care Excellence (NICE) and Google. Only the first ten pages of results provided by the Google search engine were screened.

#### Review process

All citations identified through PubMed/MEDLINE and EMBASE were downloaded into reference management software and duplicates were removed. All titles and abstracts were screened by two independent reviewers (VNP, MJR), using the following standardized inclusion criteria: (a) all review articles related to maternal sepsis or identification criteria published between 2010 and 2016; (b) all articles that reported on the development/testing of identification criteria for maternal sepsis cases. The period of publication was determined to ensure that definitions and identification criteria are those use in contemporary obstetric practice. It was expected that definitions and identification criteria published before 2010 would have been included in the existing reviews identified in our search strategy.

Full-texts of the reviews with potentially relevant information on maternal sepsis, or when title/abstract was deemed insufficient for decision on inclusion/exclusion, were obtained. Full texts of potential eligible articles were assessed independently by two reviewers (VNP, MJR). All WHO documents and guidelines reporting a definition or identification criteria for maternal sepsis were assessed, independently of publication date. WHO documents and guidelines were assessed by one reviewer (MB). Data were extracted using a standardized spreadsheet specifically developed for this review and including the following domains: ‘first author’s name’; ‘year of publication’; ‘study design’; ‘definition of maternal sepsis provided’; ‘list of identification criteria of maternal sepsis provided’. Data were extracted once and extraction forms were double checked by a second reviewer. Discrepancies on inclusions and/or data extraction were resolved through discussion or, if required, by a third reviewer.

#### Data collection and synthesis

The definitions of maternal sepsis reported by the included studies, WHO documents and guidelines were extracted. Identification criteria were organized based on organ system involved (circulatory, respiratory, central nervous, renal, coagulation, digestive, skin, genital, and inflammatory). Information concerning specific clinical values or general symptoms and signs was also extracted. No meta-analysis was performed due to the non-numerical nature of the data and heterogeneity.

### Expert consultation

Based on the systematic review presented in this paper and the recently published Sepsis-3 consensus definition [1], a technical group developed draft definitions of maternal sepsis and proposed a two-stage approach to identify women with possible severe maternal infection who may benefit from prompt clinical action (e.g. further clinical or laboratory investigation, initiation of therapy, and referral), and a second set of criteria to confirm diagnosis of maternal sepsis. The results of the systematic review showed important differences in terms of variables and thresholds use to define maternal sepsis, as shown in Table 2. So it was difficult to define the variables and the lower and upper limits to be considered as suggestive of dysregulated response to infection. Therefore, the technical group decided to present two set of questions in the survey. One based on the SOFA score and a second one taking into account a recently published systematic review on physiologic parameters during pregnancy and intermediate cutoffs points [3]. Upper and lower limits used in different early warning scores not included in this review were checked to ensure consistency with current clinical practice.

An online survey was carried out to assess expert opinion on the new definition of sepsis proposed for the adult population (SEPSIS-3) and the alternatives developed by the technical group, the applicability for the obstetric population and the merits of various criteria for the identification of maternal sepsis.

The survey was sent in March 2016 to 231 experts selected from professional societies of obstetrics, midwifery, nursing and intensive care, research groups working on maternal morbidity and authors of reviews identified in the systematic review described above. Two reminders were sent to all participants during one month period. The survey was anonymous and structured with multiple choice and open-ended questions. Participants were asked to choose a preferred definition of maternal sepsis among various alternatives drafted by the technical group. The respondents were also asked to provide their level of agreement with the definition of organ dysfunction in adults and its application to maternal sepsis (in a five-point Likert scale ranging from strongly disagree to strongly agree with the statement,, and to rank the variables to be used as identification criteria. Combined percentages were calculated for the responses “Strongly agree”and “Agree”, “Strongly disagree”and “Disagree”, “Important” and “Very important”, “Not important” and “Slightly important”.

In April 2016, WHO’s Department of Reproductive Health Research and Jhpiego convened a consensus development face-to-face expert meeting, including some of the experts who participated in the online survey, to review results of this systematic review and the online consultation, and to agree on a consensus definition for maternal sepsis.

## Results

### Systematic review

**Fig. 1:**
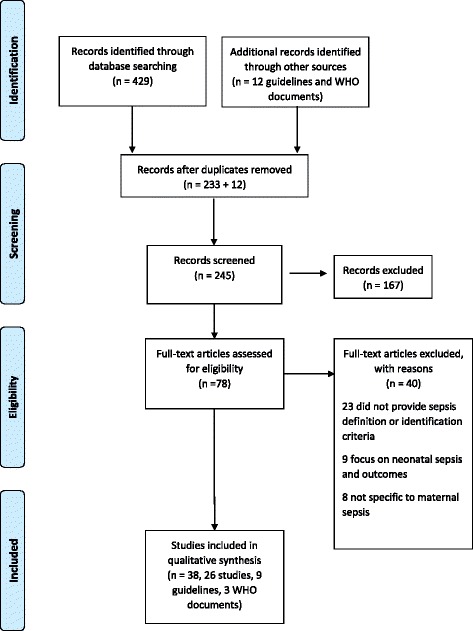
Flow chart

The search yielded to 245 citations after exclusion of duplicates. A total of 78 were selected for full-text evaluation. After screening, 26 studies [2, 4, 5, 8, 10, 12, 20–39], 9 guidelines [13, 40–47] and 3 WHO documents [48–50] matched the inclusion criteria (Fig. 1).

This review found several definitions and sets of criteria being used to identify maternal sepsis cases (Table 1 and 2). However, a substantial proportion of studies (11/26) and guidelines (7/9) referred to the First (1991) or Second (2001) International Consensus on Sepsis developed by a joint task force of American and European societies of intensive care medicine (Table 1). These international consensuses published in 1992 [17] and 2003 [18], proposed definitions and criteria for the identification of sepsis cases in the general population and were based on the previous understanding of sepsis as an infection with a systemic inflammatory response (SIRS).

**Table 1:** Summary of definitions used to refer to maternal sepsis in studies, guidelines and WHO documents included in the systematic review

Name of organization proposing the definition, (year)	Definition	Studies referring to the definition	Guidelines referring to the definition
American College of Chest Physicians and the Society of Critical Care Medicine (1992) [17]	*Systemic inflammatory response syndrome (SIRS):* Widespread inflammatory response to severe clinical insult defined by the presence of two or more of the following symptoms: ▸ Temperature >38 °C or <36 °C ▸ Heart rate >90/min ▸ Respiratory rate >20/min or PaCO_2_ < 32 mmHg ▸ White blood cells >12 × 10^9/dL or <4 × 10^9/dL or >10% immature forms *Sepsis*: SIRS plus definitive evidence of infection *Severe sepsis*: Sepsis with signs of organ dysfunction, hypoperfusion or hypotension *Septic shock*: Sepsis with hypotension despite adequate fluid resuscitation	Barton, 2012 [10]; Buddeberg, 2015 [4]; Chebbo, 2016 [22]; Cordioli, 2013 [23]; Frise, 2015 [26]; Morgan, 2013 [5]; Oud [35] Pacheco, 2014 [36]; van Dillen, 2010 [12]	FLASOG 2013 [47]
International Classification of Diseases, Revision 10 (ICD-10) (1994)	A temperature rise above 100.4 F (38 °C) maintained over 24 h or recurring during the period from the end of the first to the end of the 10th day after childbirth or abortion.	Acosta, 2013 [2]; Bamfo, 2013 [21]; van Dillen, 2010 [12]; Hashmi, 2014 [29]; Sung, 2011 [39]	-
SCCM/ESICM/ACCP/ATS/SIS International Sepsis Definitions Conference 2001 (2003) [18]	Refers to American College of Chest Physicians and the Society of Critical Care Medicine (1992) definition as above with expanded list of criteria.	Arulkumaran, 2013 [20]; Hashmi, 2014 [29]	CEC 2015 [45]; HSE 2014 [43]; NICE 2016 [42]; South Australian Perinatal Practice Guidelines 2014 [44]; RCOG 2011 [41]; RCOG 2012 [13]
The prevention and management of puerperal infections World Health Organization (WHO) (1992) [48]	*Puerperal sepsis* is an infection of the genital tract occurring at any time between the rupture of membranes or labour and the 42nd day postpartum, in which, two or more of the following are present: pelvic pain, fever, abnormal vaginal discharge and delay in the reduction of the size of the uterus	Acosta, 2013 [2]; Buddeberg, 2015 [4]; Bamfo, 2013 [21]; Karsnitz, 2013 [30]; Lamy [31] Lucas, 2011 [33]; van Dillen, 2010 [12]; Hashmi, 2014 [29]; Sung, 2011 [39]	-
Managing Complications in Pregnancy and Childbirth: A guide for midwives and doctors WHO (2003) [50]	Infection can result in failure of the circulatory system to maintain adequate perfusion of the vital organs (shock). Suspect or anticipate shock if at least one of the following is present: fast weak pulse (110 per minute or more), low blood pressure (systolic less than 90 mmHg), sweatiness or cold clammy skin, rapid breathing (rate of 30 breaths or more), anxiousness, confusion or unconsciousness, scanty urine output (less than 30 ml per hour), other symptoms and signs of shock include: pallor (specially of inner eyelid, palms or around mouth). Septic abortion: an abortion complicated with infection. Sepsis may result from infection if organisms rise from the lower genital tract following either spontaneous or unsafe abortion. Symptoms and signs are: lower abdominal pain, rebound tenderness, tender uterus, prolonged bleeding, malaise, fever, foul-smelling vaginal discharge, purulent cervical discharge, cervical motion tenderness	-	-
Midwifery education modules – Managing puerperal sepsis (WHO) (2008) [49]	*Puerperal sepsis* is any bacterial infection of the genital tract which occurs after the birth of a baby. The following symptoms and signs occur in puerperal sepsis: fever (temperature of 38 °C or more), chills and general malaise, lower abdominal pain, tender uterus, subinvolution of the uterus, purulent, foul-smelling lochia. Symptoms and signs that may also be present: light vaginal bleeding, shock *Septic abortion*: An abortion (loss of pregnancy during the first 22 weeks) that is followed by infection of the uterus and may spread throughout the genital tract causing fever and chills, foul-smelling vaginal discharge, pelvic pain and septicaemia	-	-

*ACCP* American College of Chest Physicians, *ATS* American Thoracic Society, *ESICM* European Society of Intensive Care Medicine, *SCCM* Society of Critical Care Medicine, *SIS* Surgical Infection Society

**Table 2:** List of criteria used to identify maternal sepsis in studies and guidelines included in the systematic review

System	Variable	Values or ranges (min - max)	References studies	References guidelines	Other signs	Other symptoms
Circulatory	Heart rate	>90/min (90–120/min) or > 2 SD above the normal value for age	[2, 4, 5, 10, 12, 20–23, 26, 28–31, 33, 35, 36]	[13, 40, 42–46]	Vasoplegia [34]	-
	Blood pressure	SBP <100 mmHg MAP <70 (65–70) mm Hg Decrease SBP >40 mmHg	[4, 12, 20, 22, 23, 28, 30, 31, 35, 36]	[13, 40, 41, 44–46]	Significant oedema/positive fluid balance [12, 20, 23, 35, 36, 43, 47]	-
Respiratory	Respiratory rate	>20/min (≤10 or ≥ 25/min)	[2, 4, 5, 10, 12, 21, 26, 29, 31, 35–37]	[13, 40, 42, 44–46]	Marked tachypnea, Acute lung injury in the absence of pneumonia as infection source [12, 22, 29, 36, 38]	Cough, sore throat [4, 25], chest pain [41]
	Arterial blood gases	PaO2/FIO2 <40kPa	[20–23]	[43]		
Central Nervous System	Altered mental status	Glasgow Coma scale <14/15	-	[46]	Confusion, decreased alertness, prostration, unduly anxious, distressed or panicky [4, 13, 24, 25, 40–47]	Headache, pain, lethargy, body pain, arthralgia, myalgia [4, 24, 25, 30]
	Hyperthermia	>38 °C (37.8–38.5 °C)	[2, 4, 5, 10, 12, 20–23, 26, 28–31, 33–36]	[13, 40, 43–46, 49]	-	Fever, Chills [28, 30, 33]
	Hypothermia	<36 °C (35.5 °C – 36 °C)	[2, 4, 5, 10, 12, 20–23, 26, 29–31, 33–36]	[13, 40, 43–45]	-	-
Renal	Lactate (mmol/L)	>1 (>1–4)	[8, 12, 20, 22, 23, 25, 35]	[13, 40, 42, 43, 45]	Acute oliguria, anuria [10, 12, 20, 22, 24, 25, 29, 35, 36]	Urinary symptoms [4, 25, 27], renal angle pain and tenderness [41]
	Kidney (Creat mg/dL)	>2 mg/dL or Raise > 0.5 mg/dL	[8, 12, 20, 22, 23, 25, 29, 33, 35, 37]	[41]		
Coagulation	Platelets (/ml)	<100,000	[8, 12, 20, 22, 23, 35]	[13, 40, 41, 43, 46]	-	Coagulopathy, Disseminated intravascular coagulation [8, 34, 37, 38]
		INR >1.5 or aPTT >60 s	[12, 20, 22, 23, 25, 29, 35]	[13, 40–44, 46, 47]		
Digestive	Glucose (mmol/L)	>7.7 in the absence of DM	[12, 20, 22]	[13, 40, 43, 44]	Mild intestinal tenderness without rebound tenderness Peritonitis [4, 25, 27, 30] Ileus [12, 20, 22, 23, 35, 36]	Reduced appetite, Nausea, Vomiting, diarrhea, abdominal pain [4, 10, 25, 27, 31]
	Liver: total bilirubin (mmol/L)	>2 mg/dL >70 mmol/L	[8, 12, 20, 22, 23, 25, 29, 35]	[13, 40–44, 47]		
Skin	-	-	-	-	Decreased capillary refill, clammed or mottled skin Generalized erythematous macular rash, Rash, erythema [4, 28, 38] Necrotizing fasciitis or myositis Gangrene [8, 10, 12, 20, 22, 23, 27, 35–38]	-
Genital	-	-	-	-	Pus or foul-smelling fluid from the cervix, pyometra, cervicitis, metrorrhagia, delay in the rate of reduction of size of uterus [10, 21, 24, 27, 28, 30, 31]	Purulent vaginal discharge [30, 31] pelvic pain, [10, 21, 24, 27, 28, 30]
Inflammatory	WBC(cells/μl)	>12000 (11000–16000) or <4000 or >10% immature forms	[2, 4, 5, 10, 12, 20–23, 25, 26, 28–30, 33–36]	[13, 40–47]	Preterm labour [25]	Breast redness, Pain of site of infection [4, 10, 22, 25]
	CRP	>7 mg/L >2SD	[12, 20, 22, 23, 29, 35]	[13, 40–44, 47]		
	Procalcitonin	>2SD	[20, 22, 23, 35]	[43, 47]		
Other	Culture	Positive	[8, 10, 24, 32, 38]	-	-	-
	Fetal distress	-	-	-	Fetal distress secondary to maternal acidosis [45]	Reduced or absent fetal movements [45]

*CRP* C-reactive protein, *FIO2* fraction of inspired oxygen, *MAP* Mean Arterial Blood Pressure, *PaO2* arterial oxygen partial pressure, *SBP* Sistolic Blood Pressure, *WBC* White Blood Cell Count

Nine studies (9) referred to a previous WHO definition of “puerperal sepsis” as an infection of the genital tract occurring at any time between the onset of rupture of membranes or labour and the 42nd day postpartum, without any reference to complicated infections and systemic response. Another WHO document [49] gives a slightly different definition of puerperal sepsis as any bacterial infection of the genital tract which occurs after the birth of a baby. The most recent WHO documents [49, 50] also provides a definition of septic abortion as an abortion complicated with infection of the genital-tract. However these two documents also included shock or septicemia as one of the clinical presentations that may also occur with infection during the puerperium or after an abortion.

Coverage of the different stages of pregnancy and the postpartum/post-abortion periods is not consistent. The majority of reviews and guidelines cover all stages of pregnancy, intrapartum, postpartum and post-abortion periods. However some studies [2, 4, 8, 20, 22, 30, 37] and guidelines [40, 48, 49] were limited to certain stages (e.g. postpartum) or conditions (abortion) [2, 20, 24, 31]. Furthermore, time limits for the postpartum period varied to up to the end of the 10th day after childbirth or abortion, to the 42nd day postpartum in ICD-10.

There is also variation regarding the type of infection considered. Nine of the reviews referred to any pregnancy or non-pregnancy-related infection, including incidental infections. Other preferred to differentiate those infections from nosocomial infections, or to classify infections into those related to the genital-tract infections and non-genital-tract infections. Furthermore, four documents (2 reviews [4, 30], 2 WHO documents [48, 49]) limited to postpartum genital tract infections. Three reviews focused on Group A *Streptococcus* (GAS) infections [8, 28, 38].

Criteria proposed to identify maternal sepsis also vary widely. The most common variables were temperature, heart rate, blood pressure, respiratory rate and leukocytosis. Reviews and guidelines considering laboratory findings for identification of septic women include several testing of liver (enzymes, bilirubin), kidney (creatinine) and clotting (INR, aPPT) function. Five reviews [8, 10, 24, 32, 38] recommended use of cultures to identify causative microorganism, including two reviews focusing on GAS infection [8, 38] and requiring isolation of the pathogen for confirmation of diagnosis. Most of the guidelines also referred to the use of glucose, CRP and plasma procalcitonin. These variables were less frequently proposed in the included reviews. The full set of results of the systematic review is presented in Additional file 2: Tables S2 and S3.

### Expert consultation

The online survey of experts elicited 48 responses. The full set of results of the online survey is presented in Additional file 3: S4. The background of the participants was obstetrics (*n* = 23), midwifery (3), adult or obstetric critical care (10), infectious disease (3), researchers (17) and public health specialists (1), from both public (43) and private (5) sector, and representing all regions (Africa (9), Americas (14), Asia (7), Europe (11), Middle East (2).

The majority of experts stated that the definition of organ dysfunction used in maternal sepsis should be adapted from the SEPSIS-3 consensus for adults (33/46), and should be based on specific criteria for the obstetric population (35/46). Most of the respondents stated that the assessment of organ dysfunction should include respiratory, cardiovascular, renal and neurologic variables (43, 44, 41 and 38 respondents respectively out of 46 respondents to the questions). Less than half considered it important to include fetal variables (22/46). Regarding specific variables, experts tended to prefer thresholds adapted to the obstetric population over those of the qSOFA (*respiratory rate ≥22, systolic blood pressure ≤100 mmHg, and altered mentation*). Experts also preferred the use of clinical signs over laboratory investigations necessary to calculate the SOFA score. However, two-thirds of the respondents considered serum creatinine (31/44), serum lactate (29/44) and arterial pH (30/44) as important markers of clinical severity. Fourteen of these experts also participated in the face-to-face meeting, where all findings were reviewed and discussed.

## Consensus definition of maternal sepsis

As a result of this systematic review and international expert consultation, and using an iterative process to achieve convergence, a standard definition of maternal sepsis was proposed as shown in Table 3 [51]. The post-partum or post-abortion period is 42 days of termination of pregnancy.

**Table 3:** Maternal sepsis consensus definition

“Maternal Sepsis is a life-threatening condition defined as organ dysfunction resulting from infection during pregnancy, childbirth, post-abortion, or post-partum period*”*	

It is proposed that the identification of organ dysfunction takes place in two steps, one for identification of women with possible severe maternal infections, and presenting with early signs of infection with systemic repercussion (to allow initiation of timely treatment) and another for “confirmed” cases of maternal sepsis (to enable comparative studies). The attributes of these identification criteria were also agreed by the experts. These criteria should be actionable, simple to obtain, able to predict early signs of sepsis, based on clinical signs at bedside, allow for additional tests where available, and suited to high- and low-resource settings. They have to be useful in clinical practice to identify women who may benefit from early intervention. They also have to be compatible with the current classification of disease ICD-10 and contribute to the ongoing revision of the classification to allow comparability of data.

## Discussion

This paper describes the process towards a consensus definition of maternal sepsis, based on a systematic review and international expert consultation. This new WHO definition of maternal sepsis may facilitate a better understanding of the burden of this condition and allow comparisons across different settings. Ultimately this will provide the basis for effective prevention, identification and management of maternal sepsis.

The systematic review showed a wide range of definitions and criteria being used to describe maternal sepsis. Most of the reviews and guidelines identified align with the previous definitions of sepsis, severe sepsis and septic shock, and criteria used to identify adult septic patients. However, in obstetrics, the term sepsis is often used interchangeably to refer to puerperal infectious morbidity. There are also differences in the type of infections considered. Some definitions included only infections from the genital tract (chorioamnionitis, endometritis), while others have a broader definition and include infections from other organ systems, such as pneumonia, or incidental infections (malaria). The majority of studies reported maternal sepsis across all the spectrum of pregnancy, including antenatal, intrapartum, postpartum, and abortion related sepsis. All these variations may have important implications for clinical care and potentially lead to misdiagnosis, inadequate treatment or delays in care. They also have epidemiological implications, making it difficult to assess the real burden of maternal sepsis and allow comparisons across different settings (hospitals, countries).

In February 2016 the Third International Consensus on Sepsis was published and included a substantial change in the understanding of sepsis [1]. This Sepsis-3 consensus definition defined sepsis as a life-threatening organ dysfunction caused by a dysregulated host response to infection. The significance of the systemic inflammatory response was de-emphasized compared to the previous definition based on the SIRS criteria. The SOFA score was also introduced to better describe severity of organ dysfunction and to predict in-hospital mortality [1]. This scoring includes the evaluation for the following organ systems: Respiration (PaO2), Coagulation (platelets), Liver (bilirubin), Cardiovascular (mean arterial blood pressure, MAP), Central Nervous System (Glascow Coma Scale), Renal (serum creatinine and urine output).

The proposed new WHO definition of maternal sepsis aligns with the current understanding of sepsis in adults, and considers sepsis as a consequence of a dysregulated, life-threatening response to infection. This is supported by the results of the expert consultation, but also previous guidelines which already were recommending the use of definitions and criteria developed for the general population. However, many of the variables used to calculate the SOFA score were not mentioned in the included reviews or supported by results of the expert consultation. This is despite the fact that the SOFA score variables and its clinical proxies have been previously proposed by WHO as gold-standard for severity markers to be used to identify women with life-threatening conditions [52]. For example PaO2 was mentioned in three reviews only [20, 22, 23]. Glasgow Coma scale was mentioned in one of the included reviews and in one guideline only [46]. Results of the online survey showed similar results, as most of the experts preferred to use clinical markers of severity over laboratory investigation necessary to compute the SOFA score. The exception was the use of serum creatinine and lactate. These results may be in part influence by the criteria set in the survey, such as ensuring applicability in low resource settings, in a wide range of clinical settings and by different cadres of health care providers. The results also suggest that the list of identification criteria used for maternal sepsis must accommodate different resources available to clinicians, whilst allowing them to incorporate into the diagnosis laboratory findings when available.

The new definition also aligns with the WHO definition of maternal morbidity defined as “any health condition attributed to and/or complicating pregnancy and childbirth that has a negative impact on the woman’s wellbeing and/or functioning” [53]. Regarding infectious morbidities, the definition of pregnancy related infections includes genital tract infections (e.g. chorioamnionitis and endometritis), extra-genital infections (e.g. mastitis, breast abscess, pyelonephritis, tetanus), as well as other maternal infections complicating pregnancy, childbirth and the puerperium (e.g. HIV, malaria, STIs, other fungal and viral infections).

A clear problem for using clinical criteria for pregnant women is that physiological changes in pregnancy may affect many organ systems: blood pressure, PaO2, bilirubin or creatinine measurements [3]. Furthermore, experts report that clinical criteria must be applied with great care in the obstetric patient where, sometimes, only one or even none of the above findings are present despite a severe infection [4]. For this reason modified obstetric early warning scoring systems (MOEWS) have been developed for pregnant women [54]. MOEWS systems vary widely in terms of alert thresholds, format, and accuracy. However, none of the current existing tools have shown good performance in predicting the development of severe sepsis [54, 55]. A limitation of this review is that we did not include these MOEWS or SOS (Sepsis in Obstetrics) scores as potential sources of criteria for identification of septic women. However, these scores have not been prospectively validated in a large obstetric population across different settings, particularly in low- and middle-income countries where their impact could be the largest.

## Conclusion

The new maternal definition and set of identification criteria will be tested and validated in a large global study [56]. A global one-week cross-sectional study will be carried out in 2017 in a large network of health facilities, the *Global Maternal Sepsis Study*. This study aims to cover existing knowledge gaps. It will assess the frequency of maternal sepsis using the proposed definition and identification criteria in all settings, particularly in low- and middle-income countries, and across the spectrum of pregnancy including the antenatal, peripartum, postpartum and post-abortion periods. Information on prevention and management strategies will be collected to inform the development of a strategy for reducing maternal infections and sepsis.

## Additional files

Additional file 1: Table 1. Search strategy. (DOCX 16 kb)

Additional file 2: Tables 2A and B. Summary of studies included in the systematic review: definitions and identification criteria for maternal sepsis. **Tables 3A and B.** Summary of guidelines included in the systematic review: additional symptoms and signs. (DOCX 115 kb)

Additional file 3: Table S4. Results of the online survey. (DOCX 74 kb)

## Abbreviations

ACCP: American college of chest physicians
aPPT: Activated partial thromboplastin time
ATS: American thoracic society
CRP: C-reactive protein
ESICM: European society of intensive care medicine
GAS: Group A streptococcus
G-I-N: Guidelines international network
ICD-10: International classification of disease, version 10
INR: International normalized ratio
MAP: Mean arterial blood pressure
MOEWS: Modified obstetric early warning scoring systems
NICE: National institute for health and care excellence
PaO2: Oxygen partial pressure
qSOFA: Quick SOFA
SBP: Sistolic blood pressure
SCCM: Society of critical care medicine
SIS: Surgical Infection Society
SOFA: Sequential [Sepsis-related] organ failure assessment (SOFA) score
SOS: Sepsis in obstetrics score
WBC: White blood cell count
WHO: World health organization
