# Correction to: Towards a consensus definition of maternal sepsis: results of a systematic review and expert consultation

**DOI:** 10.1186/s12978-018-0451-5

**Published:** 2018-01-08

**Authors:** Mercedes Bonet, Vicky Nogueira Pileggi, Marcus J. Rijken, Arri Coomarasamy, David Lissauer, João Paulo Souza, Ahmet Metin Gülmezoglu

**Affiliations:** 1 UNDP/UNFPA/UNICEF/WHO/World Bank Special Programme of Research, Development and Research Training in Human Reproduction (HRP), Department of Reproductive Health and Research, World Health Organization, Geneva, Switzerland; 2 Department of Social Medicine and Department of Paediatrics, Ribeirão Preto Medical School, University of São Paulo, Ribeirão Preto, SP Brazil; 3 Department of Obstetrics and Gynaecology and Julius Global Health, Julius Center for Health Sciences and Primary Care, Utrecht University Medical Centre, Utrecht, The Netherlands; 4 Institute of Metabolism and Systems Research, University of Birmingham, Birmingham, UK; 5 Birmingham Women’s National Health Service (NHS) Foundation Trust, Birmingham, UK; 6 Birmingham Centre for Women’s and Children’s Health, College of Medical and Dental Sciences, University of Birmingham, Birmingham, UK

## Correction

After publication of the original article [1], it came to the authors’ attention that the Acknowledgements section was not completed correctly. The Acknowledgements of the article should have been as follows.
