# Supplementary material for: Towards a consensus definition of maternal sepsis: results of a systematic review and expert consultation
Source: Reprod Health. 2017 May 30;14:67. doi: 10.1186/s12978-017-0321-6 (PMC5450299; doi:10.1186/s12978-017-0321-6)
Supplement: Supplementary file 1 — Search strategy. (DOCX 16 kb) [file 12978_2017_321_MOESM1_ESM.docx]

**Supplementary Table S1. Search strategies**

| **Search** | **Search strategy PUBMED** | **Citations found in the last 5 years (all time)** | **Retrieved papers** |
| --- | --- | --- | --- |
| #1 | “maternal sepsis” | 47 (84) | 14 |
| #2 | (maternal septic shock) NOT ("Maternal Sepsis") | 62 (211) | 10 |
| #3 | ((Mother OR Maternal OR Pregnancy OR Childbirth OR Postpartum OR Puerperal) AND (Sepsis OR Septicemia)) AND (death OR mortality OR near miss OR severe morbidity OR critical care OR Intensive Care OR ICU OR shock OR emergency OR critical illness) NOT (Neonatal Sepsis OR Newborn Sepsis) NOT (maternal septic shock) NOT ("Maternal Sepsis") | 505 (2155) | 53 |
| #4 | ((Mother OR Maternal OR Pregnancy OR Childbirth OR Postpartum OR Puerperal) AND (Sepsis OR Infection)) AND (definition OR identification criteria OR diagnostic criteria) | 726 (2447) | 157 |
|  | **Total** | **1340 (4897)** | **234** |

| **Search** | **Search strategy** EMBASE | **Citations found in the last 5 years (all time)** | **Retrieved papers** |
| --- | --- | --- | --- |
| #1 | “maternal sepsis” | 88 (125) | 13 |
| #2 | (maternal septic shock) NOT ("Maternal Sepsis") | 171 (345) | 25 |
| #3 | ((Mother OR Maternal OR Pregnancy OR Childbirth OR Postpartum OR Puerperal) AND (Sepsis OR Septicemia)) AND (death OR mortality OR near miss OR severe morbidity OR critical care OR Intensive Care OR ICU OR shock OR emergency OR critical illness) NOT (Neonatal Sepsis OR Newborn Sepsis) NOT (maternal septic shock) NOT ("Maternal Sepsis") | 1072 (1729) | 108 |
| #4 | ((Mother OR Maternal OR Pregnancy OR Childbirth OR Postpartum OR Puerperal) AND (Sepsis OR Infection)) AND (definition OR identification criteria OR diagnostic criteria) | 399 (690) | 59 |
|  | **Total** | **1730 (2889)** | **195** |
