# Supplementary material for: Towards a consensus definition of maternal sepsis: results of a systematic review and expert consultation
Source: Reprod Health. 2017 May 30;14:67. doi: 10.1186/s12978-017-0321-6 (PMC5450299; doi:10.1186/s12978-017-0321-6)
Supplement: Supplementary file 3 — Results of the online survey. (DOCX 74 kb) [file 12978_2017_321_MOESM3_ESM.docx]

**Supplemetary file S4. Results of the expert consultation on the definition and identification criteria of maternal sepsis**

**Section 1**

1. **
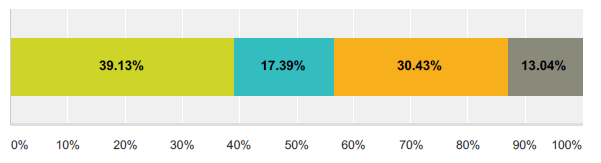
Please select your preferred definition for maternal sepsis from the proposed alternatives below:**

- Maternal sepsis is a life-threatening organ dysfunction caused by an infection that occurs during pregnancy, childbirth, postpartum, abortion or post-abortion.
- Maternal sepsis is a life-threatening organ dysfunction caused by an infection attributed to pregnancy, including antepartum, intrapartum, postpartum, abortion or post-abortion periods.
- Maternal sepsis is a life-threatening organ dysfunction caused by a dysregulated host response to infection during pregnancy, childbirth, postpartum, abortion or post-abortion.
- Maternal sepsis is a life-threatening organ dysfunction caused by a dysregulated host response to infection attributed to pregnancy, including antepartum, intrapartum, postpartum, abortion or post-abortion periods.

1. **
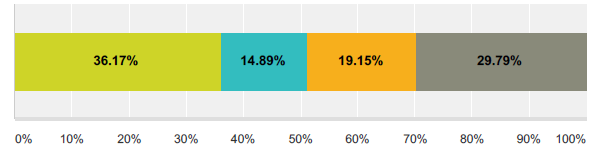
 Please select your preferred definition for suspected maternal sepsis from the proposed alternatives below**

- Suspected maternal sepsis is a suspected or confirmed infection during pregnancy, childbirth, postpartum, abortion or post-abortion with clinical signs of organ dysfunction.
- Suspected maternal sepsis is a suspected or confirmed infection attributed to pregnancy, childbirth, postpartum, abortion or post-abortion with proxy indicators of organ dysfunction.
- Suspected maternal sepsis is a presumed case of organ dysfunction in the presence of suspected or confirmed infection during pregnancy, childbirth, postpartum, abortion or post-abortion.
- Suspected maternal sepsis is a potentially life-threatening condition associated with infection (suspected or confirmed) during pregnancy, childbirth, postpartum, abortion or post-abortion.

**Section 2**

**Instructions:** When responding to questions in section 2, please consider to tie your answer to the following principles for the development of identification criteria:

- Identification criteria should be **applicable in low resource settings** (useful in any setting regardless of the development status)

- Identification criteria should be **applicable in different clinical settings**, including out-of-hospital (community), emergency department, hospital ward, labour ward, ICU and **by different health care** **providers** (nurses, skilled-birth attendants, midwives, general doctors, obstetricians, anaesthesiologist, etc)

- Identification criteria should be **useful for management of individual patients in clinical practice** (allow early management, application of bundles of care, trigger appropriate referral if needed) and patient monitoring (allow repeated assessment of clinical presentation)

1. **Please rate your level of agreement with each of the following statements**

The definition of organ dysfunction used in maternal sepsis

should be consistent with the SEPSIS-3 consensus (SOFA

score=2)* for adults

The definition of organ dysfunction used in maternal sepsis

should be adapted from the SEPSIS-3 consensus for adults

The definition of organ dysfunction used in maternal sepsis

should be based on specific criteria for the obstetric population

1. **The definition of organ dysfunction should include parameters in the following organ systems variables:**
2. **What are in your opinion the variables that should be considered when developing a list of identification criteria for suspected maternal sepsis**
3. **What are in your opinion the variables that should be considered when developing a list of identification criteria for confirmed maternal sepsis**

**Clinical variables: vital signs**

**Other clinical variables**

**Laboratory tests**

**Management variables**
