# Supplementary material for: Towards a consensus definition of maternal sepsis: results of a systematic review and expert consultation
Source: Reprod Health. 2017 May 30;14:67. doi: 10.1186/s12978-017-0321-6 (PMC5450299; doi:10.1186/s12978-017-0321-6)
Supplement: Supplementary file 3 [file 12978_2017_321_MOESM2_ESM.docx]

Supplementary table 2A. Summary of studies included in the systematic review: definitions and identification criteria for maternal sepsis

First Author (Year of Publication)	Sepsis definition used	Scope	Infections included	Temperature (ºC)	Heart Rate (bpm)	Blood pressure (mm Hg)	Respiratory rate (breaths/min)	Urine output	Gastrointestinal	Skin	Mental status	Other	Lab / WBC (cells/µl)	Lab/ Platelets (/ml)	/ Lactate (mmo	Lab/ LiverFT	idney F (Creat m	Lab/ Clotting	Lab/Culture	Lab/ Glucose (mmol/L)	Lab/ CRP	Lab/ other
Acosta (2013)	ICD-10, WHO	Postpartum, postabortion	Not specified	>38 or <36	>90	-	>20 or PaCO2<32 mmHg (4.3 kPa)	-	-	-	-	-	>12000 or <4000 or >10% immature forms	-	-	-	-	-	-	-	-	-
Anderson (2014)	GAS Toxic Shock Syndrome	Postpartum	-	Fever or rigors	-	SBP<90	Acute onset of diffuse pulmonary infiltrates and hypoxemia in absence of cardiac failure or by evidence of diffuse capillary leak manifested by acute onset of generalized edema	-	-	Generalized erythematous macular rash that may desquamate, Soft tissue necrosis, including necrotizing fasciitis or myositis, or gangrene	-	-	-	<100,000	-	>2 upper L	> 2 mg/dL	DIC, prolonged clotting times, low fibrinogen level, and the presence of fibrin degradation products	Isolation of group A streptococcus	-	-	-
Arulkumaran (2013)	SIRS with infection	Postpartum, postabortion	Pregnancy-related infection, Non-pregnancy-related infection, Nosocomial infections	>38.3 or <36	>90	SBP <90; MAP <70, SBP decrease >40 or > 2 SD below normal for age	Tachypnea	Acute oliguria (urine output <0.5 mL/kg/hr for at least 2 hrs despite adequate fluid resuscitation)	Ileus (absent bowel sounds)	Significant oedema; Decreased capillary refill or mottling.	Altered mental status	Arterial hypoxaemia (PaO2/FIO2 < 300) Ileus (absent bowel sounds)	>12000 or <4000 or >10% immature forms	<100,000	>1	total bilirubin >70 mmol/L	Rise >0.5	INR >1.5 or aPTT >60 s	NA	>7.7 in the absence of DM	>2 SD	procalcitonin >2 SD
Bamfo (2013)	ICD-10, WHO	Pregnancy, postpartum, postabortion	Pregnancy-related infection, Non-pregnancy-related infection	> 38 or <36	> 90	-	>20 or PaCO2< 32 mmHg	-	-	-	-	-	>12000 or <4000 or >10% immature forms	-	-	-	-	-	-	-	-	-
Barton (2012)	SIRS with infection	Pregnancy, postpartum, postabortion	Pregnancy-related infection, Non-pregnancy-related infection	> 38 or <36	> 90 severe >110	Hypotension or shock	> 20 or PaCO2< 32 mm Hg, severe >24	Oliguria or anuria		Clammy or mottled skin; Diaphoresis	Altered mental state (confusion, decreased alertness)	Hypoxemia; Disseminated intravascular coagulation	>12000 or <4000 or >10% immature forms	Thrombocytopenia	Increased; Low arterial pH; Increased base deficit)	Elevated liver enzymes	Elevated serum creatinine	-	Positive culture from infection site or blood, or infection site and blood	Hyperglycemia in the absence of diabetes	-	-
Buddeberg (2015)	SIRS, WHO	Postpartum	Genital-tract infections	> 38.5 Fever or rigours	>90	-	>20 or PaCo2 <32 mm Hg	Urinary symptoms	Diarrhoea or vomiting	Rash;	-	-	>12000 or <4000 or >10% immature forms	-	-	-	-	-	-	-	-	-
Chebbo (2016)	SIRS	Pregnancy, postpartum	Pregnancy-related infection, Non-pregnancy-related infection	>38.3 or <36	>90 or more than 2 SD above the normal value for age	SBP <90; MAP <70, SBP decrease >40 or > 2 SD below normal for age	Tachypnea; Arterial hypoxemia (PaO2/FiO2 <300)	Acute oliguria (urine output <0.5 mL/kg/h for at least 2 h despite adequate fluid resuscitation)	Nausea/vomiting; Ileus (absent bowel sounds)	Decreased capillary refill or mottling; Significant edema or positive fluid balance (>20 mL/kg over 24 h); Clammy/mottled skin;	Altered mental status;(confusion, decreased alertness)	-	>12000 or <4000 or >10% immature forms	<100,000	>1	total bilirubin >70 mmol/L	Rise >0.5	INR >1.5 or aPTT >60s	NA	>7.7 in the absence of DM	> 2 SD above the normal value	Plasma procalcitonin > 2 SD
Cordioli (2013)	SIRS	Pregnancy, postpartum, postabortion	Pregnancy-related infection, Non-pregnancy-related infection, Incidental infections, Nosocomial infections	>38,3 or <36	>90 or more than 2 SD above the normal value for age	SBP <90; MAP <70, SBP decrease >40 or > 2 SD below normal for age	Taquypnea, PaO2/FiO2 <300	<0,5mL/kg/h	Ileus (absent bowel sounds)	Significant oedema ; decreased capillary refill or mottling	Altered mental status	Cardiac index >3,5L/min/m2;Central venous oxygen saturation (ScvO2) >70%	>12000 or <4000 or >10% immature forms	<100,000	>3	total bilirubin >70 mmol/L	Rise >0.5	INR >1,5 or aPTT >60 s	-	-	>2	Plasma procalcitonin > 2 SD
Eschenbach (2015)	septic abortion diagnosis	Postabortion	-	NA	marked tachycardia	Hypotension	marked tachypnea with or without respiratory difficulty,	Low urine output	-	-	-	-	-	-	-	-	-	-	Aerobic and anaerobic cultures of the cervix, products of conception (if present), or both	-	-	-
Ford (2014)	No	Pregnancy, postpartum, postabortion	Not specified	Pyrexia, hypothermia	Tachycardia	Hypotension	-	Oliguria	Diarrhea, vomiting,	-	Impaired consciousness	-	>12000 or <4000	-	>4	-	-	increase INR APTT	-	-	Increase	calcitonin rise

Frise (2015)	SIRS with infection	Pregnancy, postpartum, postabortion	Not specified	< 36 or > 38	> 90	Hypotension	>20 (or hypocapnia below 4.3 kPa)		-		Confusion		>12000 or <4000 or >10% immature forms	-	-	-	-	-	-	-	-	-
Guntupalli (2015)	No	Pregnancy	Pregnancy-related infection, Non-pregnancy-related infection	-	-	-	-	-	-	Generalized blanching erythema (streptococcal toxic shock syndrome)	-	-	-	-	-	-	-	-	-	-	-	-
Hamilton (2013)	GAS Toxic Shock Syndrome	Pregnancy, postpartum, postabortion	-	≥38.0	≥100	SBP ≤90	-	-	-	Erythema	-	-	>11000	-	-	-	-	-	-	-	elevated	-
Hashmi (2014)	WHO, ICD-10, SIRS	Pregnancy, postpartum, postabortion	Not specified	≥ 38 or ≤ 36	≥ 90	-	>20 or PaCO2 < 32 mmHg	Decreased urine output <0.5ml/kg/hr for > 2 hrs	-		Decreased Glasgow Coma Scale score		>12000 or <4000 or >10% immature forms	-	>4	Bilirubin > 2 mg/dl	Creatinine > 2 mg/dl	INR >1.5	-	-	>2 SD	-
Karsnitz (2013)	WHO	Postpartum	Genital-tract infections	> 38 or <36 Temperature instability	>120	SBP <90; SBP decrease >40	tachypnea	-	-	-	-	-	>15000 or leukopenia / neutropenia or >10% immature forms	-	-	-	-	-	-	-	-	-
Lamy (2012)	WHO	post-abortion schock due to *Clostridium sordellii* or *C perfringens*		≥ 37,8	-	SBP<90	-	-	-	-	-	-	-	-	-	-	-	-	-	-	-	-
Lapinsky (2013)	No, clinical description of severe pregnancy related diseases	Pregnancy, postpartum, postabortion	Pregnancy-related infection, Non-pregnancy-related infection	-	-	-	-	-	-	-	-	-	-	-	-	-	-	-	Diagnosis is confirmed by Gram stain and culture of amniotic fluid.	-	-	-
Lucas (2011)	WHO	Pregnancy, postpartum, postabortion	Genital-tract infections, Non-Genital-tract infections, incidental infections	>38 or <36	>90	NA	>20 or PaCO2 < 32 mmHg		-				>12000 or <4000 or >10% immature forms	NA	NA	NA	NA	NA	NA	NA	NA	NA
Morgan (2013)	SIRS with infection	Pregnancy, postpartum, postabortion	Pregnancy-related infection, Non-pregnancy-related infection	>38 or <36	>90	-	>20 or PaCO2 <32 mm Hg		-				>12000 or <4000 or >10% immature forms	-	-	-	-	-	-	-	-	-
Neligan (2011)	No	Pregnancy, postpartum, postabortion	Pregnancy-related infection, Non-pregnancy-related infection	>38	-	-	-	-	-	-	-	-	-	-	-	-	-	Coagulopathy	-	-	-	-
Oud (2016)	SIRS with infection	Not specified	Not specified	>38 or <36 on 2 occasions >2H apart	>100 on 2 occasions >2H apart	SBP <90; MAP <70, SBP decrease >40 or <2SD below standard for age	>20 on 2 occasions >2H apart	acute oligouria	Ileus (absent bowel sounds)	Decreased capillary refill or mottling Significant edema	Altered mental status	-	>17000 or <4000 or >10% immature forms on 2 occasions >2H apart	<100,000	>1	Bilirubin > 4 mg/dl	Creatinine increase >0.5 mg/dl	INR >1.5 or aPTT >60 s	-	Hyperglycemia in the absence of diabetes	> 2 SD above the normal value	Plasma procalcitonin > 2 SD
Pacheco (2014)	SIRS with infection	Not specified	Not specified	>38 or <36	>90	SBP <90; MAP <65, SBP decrease >40	>20 or a PaCO2<32 mm Hg.	-	Ileus (absent bowel sounds)	Decreased capillary refill or mottling	Altered mental status	-	>12000 or <4000 or >10% immature forms	Thrombocytopenia	Hyperlactatemia	Hyperbilirubinemia	Acute kidney injury (acute elevation of serum creatinine 0.3 mg/dL or urine output <0.5 mL/kg/h for 6 h)	(prolonged aPTT or INR, decreased fibrinogen)	-	Hyperglycemia (not explained by other medical conditions)	-	Elevated procalcitonin levels
Sung (2011)	SIRS with infection, WHO, ICD-10	Pregnancy, postpartum, postabortion	Pregnancy-related infection, Non-pregnancy-related [illegible]	>38 or <36	>90	SBP <90; MAP <70, SBP decrease >40 or <2SD below standard for [illegible]	>20 or a PaCO2<32 mm Hg.	-	-	-	-	-	>12000 or <4000	-	-	-	-	-	-	-	-	-

Sriskandan (2011)	No	Peripartum	Genital-tract infections, Non-Genital-tract infections,	Pyrexia or hypothermia	Tachycardia	NA	>20	-	-	Blanching erythema (toxic shock)	-	-	Leucopenia or leucocytosis	Thrombocytopenia	Raised lactate/low pH	-	-	Coagulopathy	NA	NA	Raised	-
Van Dillen (2010)	SIRS with infection, WHO, ICD-10	Not specified	Pregnancy-related infection, Non-pregnancy-related infection	>38.3 or <36.0	>90 or >2 SD above the normal value for age	SBP <90; MAP <70, SBP decrease >40 or > 2 SD below normal for age	>20 or PaCO2 <32 mmHg (4.3 kPa)	Acute oliguria (urine output <0.5 ml/kg/h or 45 mmol/l for at least 2 h)	Ileus (absent bowel sounds)	Significant oedema; Decreased capillary refill or mottling	Altered mental status	-	>12000 or <4000 or >10% immature forms	<100,000	>1	total bilirubin >70 mmol/L	Creatinine increase >0.5 mg/dl	INR >1.5 or aPTT >60 s	NA	>7.7 in the absence of DM	>2 SD	procalcitonin
Yamada (2010)	GAS diagnosis Toxic Shock Syndrome	Pregnancy, postpartum, postabortion	-	-	-	SBP<90	-	-	-	-	-	-	-	<100,000	-	Bili >2 upper L	Creatinine > 2 mg/dl	DIC, prolonged clotting times, low fibrinogen level, and the presence of fibrin degradation products	Isolation of group A Streptococci (Streptococcus pyogenes)	-	-	-

Supplementary table 2B. Summary of studies included in the systematic review: additional symtomps and signs

First Author	Symptoms	Signs
Acosta (2013)	-	-
Anderson (2014)	-	-
Arulkumaran (2013)	-	Positive fluid balance (>20 ml/kg over 24 hrs)
Bamfo (2013)	Abnormal vaginal discharge (e.g. pus); abnormal smell of discharge; delay in the rate of reduction of size of uterus (less than 2 cm a day during the first 8 days).	-
Barton (2012)	Pain (location based on site of infection); Nausea or vomiting	-
Buddeberg (2015)	Pelvic pain; abnormal vaginal discharge (abnormal smell/foul odour of discharge, heavy lochia); Delay in uterine involution ;Abdominal/pelvic pain and tenderness; Productive cough; Breast engorgement/redness; Wound infection; General, lethargy, reduced appetite	Delay in the rate of reduction of the size of the uterus (<2 cm/day during the first 8 days).
Chebbo (2016)	Pain (location based on site of infection)	Disseminated intravascular coagulopathy; Hypoxemia; Acute lung injury with PaO2/FiO2 <250 in the absence of pneumonia as infection source
Cordioli (2013)	-	Positive fluid balance (>20 ml/kg over 24 hrs)
Eschenbach (2015)	Cervical or vaginal trauma, foul-smelling or purulent cervical–vaginal discharge; cervical and uterine tenderness and adnexal tenderness or mass	Prostration, Mild tenderness without rebound tenderness; general peritonitis; Products of conception remaining in the uterus; free air in the abdomen and gas in the myometrium
Ford (2014)	Vaginal discharge, headache, urinary symptoms, or productive cough. Abdominal or pelvic pain, particularly if disproportionate to that which would be expected, may indicate infection. Sore throat. Persistent constant ache between contractions (as sign of chorioamnionitisor abruption); feeling of being unwell; arthralgia and myalgia; Breast pain	Preterm labor
Frise (2015)	-	-
Guntupalli (2015)	Lower abdominal or perineal pain, vaginal discharge, frequency of urination, and diarrhea	Retained products of conception, pyometra, or abscess formation in the myometrium, parametrium, or pelvis. Intramyometrial gas pockets
Hamilton (2013)	Chills, and abdominal pain in a pregnant or early postpartum woman; Uterine tenderness; Vaginal discharge; Extremity pain	Hypotension, and tachycardia
Hashmi (2014)	-	Acute Lung Injury with PaO2/ FiO2 <300
Karsnitz (2013)	Generalized body aches and malaise; pelvic pain, abnormal vaginal discharge (unusual or foul smelling vaginal discharge or lochia), abnormal odor, or delay in involution; pelvic pain; uterine subinvolution	Chills;purulent vaginal discharge
Lamy (2012)	Abdominal pain; malodorous vaginal discharge; discomfort; cervicitis or metrorrhagia<br>Specific for schock due to *Clostridium sordellii* : refractory tachycardia, local edema and pleural effusion and peritoneal<br>Specific for schock due to *Clostridium perfringens* :hemolitci anaemia, renal failure, hemoglobinuria, jaudince	-
Lapinsky (2013)	-	-
Lucas (2011)	Pelvic pain, fever, abnormal vaginal discharge, abnormal smell of discharge, delay in postpartum reduction of size of uterus	-
Morgan (2013)	-	-
Neligan (2011)	-	Vasoplegia, and evolving multiple organ failure
Oud (2016)	-	Significant positive fluid balance (>20 mL/kg over 24 h)
Pacheco (2014)	-	Significant positive fluid balance (>20 mL/kg over 24 h); Arterial hypoxemia (PaO2/FIO2<300);
Sung (2011)	-	-
Sriskandan (2011)	-	-
Van Dillen (2010)	-	Positive fluid balance (>20 ml/kg over 24 h); SvO2 >70%; Cardiac index >3.5 l/min/m2; Arterial hypoxemia (PaO2/FiO2 <300)

Yamada (2010)	-	Adult respiratory distress syndrome infiltrates and hypoxaemia in the absence of cardiacfailure, or evidence of diffuse capillary leak manifested by acute onset of generalised oedema, or pleural or peritoneal effusions with hypoalbuminaemia<br>A generalised erythematous macular rash that may desquamate.<br>Soft-tissue necrosis, including necrotising fasciitis or myositis, or gangrene.

Supplementary table 3A. Summary of guidelines included in the systematic review: definitions and identification criteria for maternal sepsis

Guideline	Reference used for definition of sepsis	Reference used for definition of identification criteria	Scope	Comments/Clarifications	Temperature (℃)	Heart Rate (bpm)	Blood pressure (mm Hg)	Respiratory rate (breaths/min)	Urine output	Lab / WBC (cells/µl)	Lab/ Platelets (/ml)	b/ Lactate (mmol/	Lab/ LiverFT	idney F (Creat m	Lab/ Clotting	Lab/Culture	Glucose (mm	Lab/ CRP	Lab/ other
Clinical Excellence Commision (CEC)/ (Australia, 2015)	Not specified	-	Adult population. Provides a specific pathway for maternal sepsis	SEPSIS KILLS program is a quality improvement program focused on improving the recognition and management of sepsis in adults and children in the inpatient wards of public hospitals.	>38.5 or <35.5	≤ 50 or ≥ 120	< 100	≤ 10 or ≥ 25	-	NA	NA	≥ 2	NA	NA	NA	NA	NA	NA	SpO2 < 95%
Department of Health, Government of South Australia, South Australian Perinatal Practice Guidelines/ Clinical Guideline Sepsis in pregnancy (Australia, 2014)	ACCP/SCCM Consensus Conference (2001)	-	Pregnant women (all gestations) and women in the puerperium, including post termination of pregnancy/ miscarriage	The Royal College of Obstetricians and Gynaecologists Green top Guideline 64a - Bacterial sepsis in pregnancy (April 2012) has largely been used to inform this guideline	>38 or <36	>90	SBP <90; MAP <70, SBP decrease >40	> 20	-	>12000 or <4000 or >10% immature forms	NA	NA	total bilirubin >70 mmol/L	rise of >44.2µmol/l	INR >1.5 or aPTT >60 s	NA	>7.7 in the absence of DM	> 7 mg/L	NA
Federación Latino Americana de Sociedades de Ginecología y Obstetricia (FLASOG)/Guía Clínica Nº 1: Sepsis y Embarazo (Latin America, 2013)	-	Surviving Sepsis Campaign: International guidelines for management of severe sepsis and septic shock (2012)	All obstetric patients	Provides guidance on clinical management of obstetric patients with severe sepsis or septic shock in the intensive care unit or other unit	>38.3 or <36	>90 or two standard deviations for age	SBP <90; MAP <70, SBP decrease >40	Tachypnea; Arterial hypoxemia (PaO2/FiO2 <300)	Acute oliguria (urine output <0.5 mL/kg/hr for at least 2 hrs despite adequate fluid resuscitation)	>12000 or <4000 or >10% immature forms		>1	total bilirubin >70 mmol/L	rise >44.2µmol	INR >1.5 or aPTT >60 s		>7.7 in the absence of DM		Protein C > 2 SD Plasma procalciton in > 2 SD
Health Service Executive (HSE)/ Guidelines for the Critically Ill Woman in Obstetrics, (Ireland, 2014)	-	-	Patients presenting to the ED, adult in-patient, maternity patient	The aim of the guideline is to facilitate the early recognition and appropriate treatment of sepsis in Ireland in order to maximize survival opportunity and minimize the burden of chronic sequelae<br>Provides a care pathway for the deteriorated critically ill pregnant woman and a list of relevant national guidelines and reports for infection and pregnancy<br>Recommends use of the Irish Maternity Early Warning System (IMEWS) chart	>38 or <36	≥ 100	Not mentioned	Tachypnoea	-	> 16000 < 4000 or 10% immature forms	<100,000	>1	total bilirubin >70 mmol/L	rise of >44.2µmol/l	INR >1.5 or aPTT >60 s	Not mentioned	>7.7 in the absence of DM	>2 SD	Plasma procalciton in > 2 SD
National Department of Health/ Essential steps in the management of common conditions associated with maternal mortality (South Africa, 2007)	Not specified	-	Postpartum or postabortion period	Provides guidance on identification and management of severe sepsis after childbirth or abortion	> 38	>100	< 90	> 22	-	Low or high white cell count	<100,000	NA	NA	NA	Prolonged INR or PTT, Raised fibrinogen	NA	NA	NA	NA
National Institute for Health and Care Excellence (NICE) Sepsis: the recognition, diagnosis and management of sepsis: Methods, evidence and recommendations. Draft for consultation, (UK, 2016)	ACCP/SCCM Consensus Conference (2001)	-	All populations will be included, including pregnant women	This is a draft version of the document. Proposed the use of the SOS (Sepsis in Obstetrics Score for pregnant and post-partum women as scoring system)	Temperature	Heart Rate (bpm)	systolic blood pressure (mmHg)	-	-	WBC count	NA	lactic acid	NA	NA	NA	NA	NA	NA	NA
Royal College of Obstetricians and Gynaecologists (RCOG)/Maternal Collapse in Pregnancy and the Puerperium. Green–top Guideline No. 56, (UK, 2011)	ACCP/SCCM Consensus Conference (2001)	Surviving Sepsis Campaign: International guidelines for management of severe sepsis and septic shock (2008)	Pregnant women (all gestations) and women in the puerperium, in hospital and community settings	The purpose of this guideline is to discuss the identification of women at increased risk of maternal collapse and the different causes of maternal collapse, to delineate the initial and continuing management of maternal collapse and to review maternal and neonatal outcomes	>38 or <36	>100	SBP <90; MAP <70, SBP decrease >40	>20	-	>12000 or <4000 or >10% immature forms	<100,000	≥ 4	total bilirubin >70 mmol/L	rise of >44.2µmol/l	INR >1.5 or aPTT >60 s	Not mentioned	>7.7 in the absence of DM	> 7 mg/L	NA
Royal College of Obstetricians and Gynaecologists (RCOG)/Bacterial Sepsis in Pregnancy. Green–top Guideline No. 64a (UK, 2012)	ACCP/SCCM Consensus Conference (2001)	Surviving Sepsis Campaign: International guidelines for management of severe sepsis and septic shock (2008)	Pregnant women and intrapartum period	The purpose of this guideline is to provide guidance on the recognition and management of serious bacterial illness in the antenatal and intrapartum periods, arising in the genital tract or elsewhere, and its management in secondary care. Sepsis arising due to viral, fungal or other infectious agents is outside the scope of the guideline.	>38 or <36	>100	SBP <90; MAP <70, SBP decrease >40	>20 or Arterial hypoxaemia (PaO2 (arterial oxygen partial pressure) /FIO2 (fraction of inspired oxygen) <40kPa	-	>12000 or <4000 or >10% immature forms	<100,000	≥ 4	total bilirubin >70 mmol/L	rise >44.2µmol	INR >1.5 or aPTT >60 s	NA	>7.7 in the absence of DM	>7mg/l	NA
Royal College of Obstetricians and Gynaecologists (RCOG)/Bacterial Sepsis following Pregnancy. Green–top Guideline No. 64b (UK, 2012)	ACCP/SCCM Consensus Conference (2001)	Surviving Sepsis Campaign: International guidelines for management of severe sepsis and septic shock (2008) Lewis G. The Confidential Enquiry into Maternal and Child Health (CEMACH). Saving Mothers' Lives: Reviewing Maternal Deaths to Make Motherhood Safer 2003–2005. The Seventh Report on Confidential Enquiries into Maternal	Women in the puerperium after birth until 6 weeks postnatally	The purpose of this guideline is to provide guidance on the management of bacterial sepsis in the puerperium.<br>Use criteria from CEMACH where pregnancy-specific parameters available.<br>Provides clinical disease definitions for Staphylococcal and streptococcal toxic shock syndrome.	>38 or <36	>100	SBP <90; MAP <70, SBP decrease >40	>20	-	>12000 or <4000 or >10% immature forms	<100,000	≥ 4	total bilirubin >70 mmol/L	rise of >44.2µmol/l	INR >1.5 or aPTT >60 s	Not mentioned	>7.7 in the absence of DM	> 7 mg/L	NA
**WHO documents**																			
Managing Complications in Pregnancy and Childbirth: A guide for midwives and doctors World Health Organization (WHO) (2003)*	-	-	All obstetric patients	-	-	>110 per minute	SBP <90	>30	less than 30 ml per hour	-	-	-	-	-	-	-	-	-	-

*provides criteria for shock during pregancy, postpartum or post-abortion period; bmp: beats per minute; SBP: Systolic blood pressure

Supplementary table 3B. Summary of guidelines included in the systematic review: additional symtomps and signs

Guideline	Symptoms	Signs
Clinical Excellence Commision (CEC)/ (Australia, 2015)	-	altered level of consciousness or new onset of confusion, Ileus (absent bowel sounds)
Department of Health, Government of South Australia, South Australian Perinatal Practice Guidelines/ Clinical Guideline Sepsis in pregnancy (Australia, 2014)	-	altered mental status; altered level of consciousness, Fetal distress secondary to maternal acidosis
Federación Latino Americana de Sociedades de Ginecología y Obstetricia (FLASOG)/Guía Clínica Nº 1: Sepsis y Embarazo (Latin America, 2013)	-	altered mental status; significant oedema or Positive fluid balance (>20 ml/kg over 24 h), Ileus (absent bowel sounds)
Health Service Executive (HSE)/ Guidelines for the Critically Ill Woman in Obstetrics, (Ireland, 2014)	-	altered mental status; Arterial hypoxaemia (PaO2 (arterial oxygen partial pressure) /FIO2 (fraction of inspired oxygen) <40kPa)
National Department of Health/ Essential steps in the management of common conditions associated with maternal mortality (South Africa, 2007)	symtomps/signs of infection	confusion, delirium, decreased level of consciousness, Glasgow Coma scale <14/15, cold or clammy extremities
National Institute for Health and Care Excellence (NICE) Sepsis: the recognition, diagnosis and management of sepsis: Methods, evidence and recommendations. Draft for consultation, (UK, 2016)	-	-
Royal College of Obstetricians and Gynaecologists (RCOG)/Maternal Collapse in Pregnancy and the Puerperium. Green–top Guideline No. 56, (UK, 2011)	abdominal or chest pain; diarrhoea and/or vomiting; uterine or renal angle pain and tenderness; anxious or distressed;	altered mental status; altered level of consciousness; Arterial hypoxaemia (PaO2 (arterial oxygen partial pressure) /FIO2 (fraction of inspired oxygen) <40kPa), Ileus (absent bowel sounds)
Royal College of Obstetricians and Gynaecologists (RCOG)/Bacterial Sepsis in Pregnancy. Green–top Guideline No. 64a (UK, 2012)	Fever or rigors, Diarrhoea or vomiting, Rash, Abdominal /pelvic pain and tenderness, Offensive vaginal discharge, Productive cough, Urinary symptoms	altered mental status, Ileus (absent bowel sounds)
Royal College of Obstetricians and Gynaecologists (RCOG)/Bacterial Sepsis following Pregnancy. Green–top Guideline No. 64b (UK, 2012)	Fever or rigors, Diarrhoea or vomiting, Rash, Abdominal /pelvic pain and tenderness, Offensive vaginal discharge, Productive cough, Urinary symptoms	altered mental status, Ileus (absent bowel sounds)
**WHO documents**		
Managing Complications in Pregnancy and Childbirth: A guide for midwives and doctors World Health Organization (WHO) (2003)	-	anxiouness, confusion or unconssioness, pallor (specially of inner eyelid, palms or around mouth), sweatiness or cold clammy skin
